# Supplementary figures and images for: Versatile CRISPR/Cas9-mediated mosaic analysis by gRNA-induced crossing-over for unmodified genomes
Source: PLoS Biol. 2021 Jan 14;19(1):e3001061. doi: 10.1371/journal.pbio.3001061 (PMC7837743; doi:10.1371/journal.pbio.3001061)

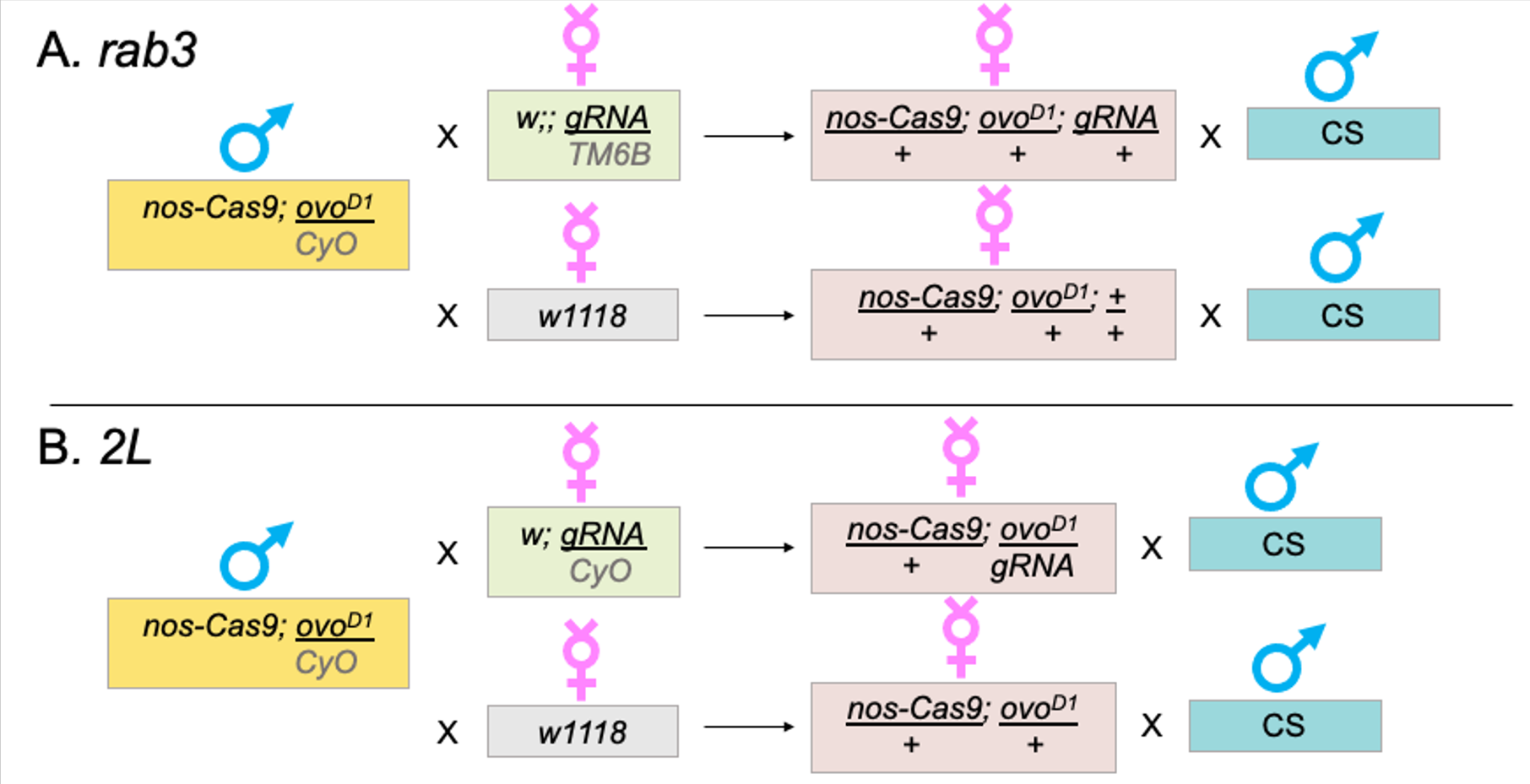

Supplement: S1 Fig — (A) Crossing scheme for germline clone induction using gRNA-Rab3, ovoD1(2R), and nos-Cas9. (B) Crossing scheme for testing gRNAs for 2L in germline clone induction. The gRNAs used in this test were gRNA(nBFP) lines. (TIF) [file pbio.3001061.s001.tif]

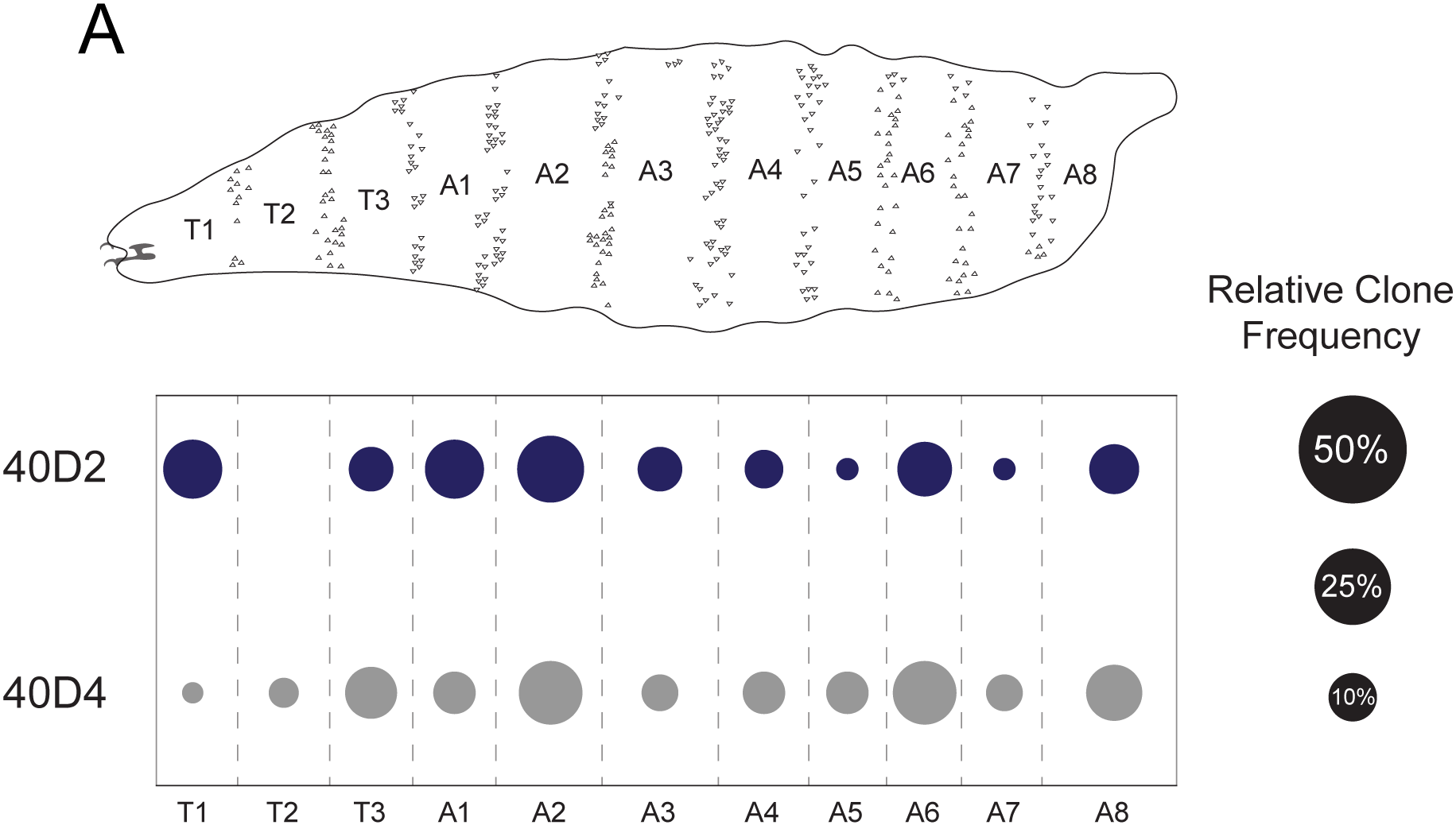

Supplement: S2 Fig — (A) Distribution of da neuron clones in each segment using gRNA(Gal80) for 40D2 and 40D4. n = number of neurons: 40D2 (n = 47); 40D4 (n = 52). The data underlying this Figure can be found in S1 Data. Larva drawn by G. T. K. (TIF) [file pbio.3001061.s002.tif]
