# Supplementary material for: Versatile CRISPR/Cas9-mediated mosaic analysis by gRNA-induced crossing-over for unmodified genomes
Source: PLoS Biol. 2021 Jan 14;19(1):e3001061. doi: 10.1371/journal.pbio.3001061 (PMC7837743; doi:10.1371/journal.pbio.3001061)
Supplement: Attachment — Submitted filename: Response to Reviewers (1).pdf [file pbio.3001061.s007.pdf]

We greatly appreciate the reviewers' helpful comments. We have addressed the issues they raised, with additional experiments and with rewriting, as suggested. We believe that the revisions have significantly improved the paper and hope that the editor and reviewers will agree and will consider our revised paper to be suitable for publication in *PLOS Biology*. The following briefly summarizes the major changes that we made; it is followed by our point-by-point responses to reviewers' comments.

Major changes in our manuscript are:

1. We demonstrated heat shock-induced clone generation using MAGIC as suggested by Reviewer #2. The results are included in new Figure 3.
2. We compared the efficiencies of clone induction between gRNA-induced crossover and FRT/Flp-mediated recombination in the wing imaginal disc using Cas9 and Flp driven by identical enhancers, based on the suggestion of Reviewer #2. The results are also included in new Figure 3.
3. We added quantification and statistical analyses of dendrite defects in mutant clones of *Sec5*, *Rab5*, and *Syx5* to show sample numbers and penetrance of the phenotypes, as suggested by Reviewer #2. The results are in new Figure 4.
4. We listed potential gRNA sequences for applying MAGIC to other major chromosome arms in Table S2. Although we were unable to establish full MAGIC toolkits for all chromosome arms within the timeframe allowed for the revision, we hope other researchers can generate their own MAGIC reagents based on these recommendations using our or their own vectors while we create full toolkits for all arms and make them publicly available.
5. We revised our figures and texts to address all issues raised by both reviewers. We also made other small changes to the manuscript to improve its readability.

POINT-BY-POINT RESPONSES TO REVIEWERS' COMMENTS

Reviewer #1:

Allen and colleagues describe a novel CRISPR/Cas9-based technique in Drosophila enabling the generation of genetic mosaics through interchromosomal recombination. The authors validate their approach in a number of somatic tissues and the female germline. The authors also provide a tool set for the genetic mosaic-based functional study of candidate genes on chromosome arm 2L. Lastly, the authors cross their transgenic lines to fly lines with wild-derived genomes and provide proof-of-principle.

Overall the authors present a potentially useful method for the study of candidate genes in genetic mosaic flies. Currently, the manuscript presents mainly proof-of-principle and the resource is still limited. While the data presentation is sound, the writing of the manuscript could be improved. At many passages the writing is not precise. The reader gets the impression that the authors slightly oversell their method, especially since CRISPR/Cas9-based clonal analysis is not new. Likewise, interchromosomal recombination to generate genetic mosaics is (as the authors nicely document in the Introduction) not a new approach. The combination of the two represents certainly an advance, but there are other methods that achieve the same result and I think the authors should pay special attention to not discredit established FRT/Flp-based methods. The authors should also tone down certain claims. Below I point out more specific points that require attention:

Response: We thank the reviewer for the thoughtful comments. We did not intend to discredit established FRT/Flp methods. They have played key roles in many discoveries in the past and are currently used for wide applications. In our view, MAGIC presents alternative options for certain applications and allows for some new applications, but it is not a replacement for all FRT/Flp technologies. We have toned down our claims as suggested by the reviewer.

1. In the Abstract, line 21 the authors state: '…and can be applied in any organism that…' The authors should say '… can in principle be applied…' They do not show any example where their method has been validated in another organism.

Response: Thanks to the reviewer for the suggestion. We changed our text.

2. In the Introduction, line 31 authors state: …Mosaic (also called clonal) analysis … this is simply wrong. Mosaic and clonal are two different things and the authors should please pay attention that the writing is precise. This is actually important throughout the entire manuscript. The authors at most places talk about 'clonal analysis' but how often do the authors really know that the labeled/mutated cells derive from one individual progenitor stem cell? In a clone, all cells are lineally related and derive from a single stem cell. The authors should, especially in such a methods paper, not confuse clonal with mosaic. Cells in a genetic mosaic can be a single clone of mutated/labeled cells in a tissue/animal, but most often mosaic simply means that labeled/mutated cells are present within a genetically distinct background. The cells in a mosaic do not need to be clonally-related. In fact the authors do not use inducible Cas9. Thus they will never know when the DSB was induced and interchromosomal recombination happened. As a consequence they also will not know if more than one event happened and in more than one progenitor stem cell.

Response: We thank the reviewer for pointing out the important distinction between mosaic analysis and clonal analysis, and we apologize for using them loosely in the original submission. As we show in the revised manuscript (Figure 3), MAGIC can be made inducible by heat shock. Therefore, in principle it can be used in clonal analysis, even though we did not specifically distinguish whether the labeled cells were derived from single stem cells in our experiments. We revised our text for a more stringent use of these terms and to better reflect the intent of our experiments.

3. Line 82 - the authors state: '…, a 50% chance exists for identical distal chromosome segments to sort into the same daughter cell, generating …' The authors should please precise their writing and explain the entire spectrum of segregation possibilities in more detail. If recombination happens in G2 phase of the cell cycle, there are two segregation possibilities, G2-X (recombinant chromosomes segregate away from each other) and G2-Z (recombinant chromosomes 'sort' together into the same cell). The authors should elaborate for the non-specialist reader and clarify the schematic in Figure 1.

Response: We thank the reviewer for this suggestion. We revised the text to better explain all outcomes of chromosome segregation illustrated in Figure 1, as suggested.

4. The authors state on line 119 - '…label clones homozygous … either negatively or positively…'. How can something be negatively labeled. Please be precise the writing.

Response: Thanks to the reviewer for pointing this error. We have changed our text to remove the phrase “negative labeling”.

5. Line 129 - '… as to avoid off-target effects…'. Can the authors estimate the probability for off target effects for the gRNA they used? A 'unique' target site does not ensure that no off-target

effects occur. More generally, off-target effects cannot be completely excluded when using MAGIC and the authors should discuss this caveat in the Discussion.

Response: We agree with the reviewer that unique target sites do not exclude off-targeting. We used published algorithms to predict the probability of off-targeting. The off-targeting scores are now included in the gRNA table (Table S2). We revised our discussion to mention potential off-targeting effects, as the reviewer suggested.

6. The authors state on line 146: '… suggests that an efficient gRNA construct for one tissue will likely perform well in other tissues also…'. Please remove such speculation or show the data with quantitative assessment. Also in the discussion line 195/6.

Response: We removed this statement.

7. The authors repetitively state that '… analogous toolkits could easily be made for any other chromosome arm…' (e.g. line 148/9 and 177/8). Well, if it is so easy, why did the authors not do it and provide a more complete resource?

Response: When we say that analogous toolkits could easily be made for other chromosome arms, we meant that making reagents for other arms is intellectually straightforward and the procedures for making them are standard molecular cloning and transgenic steps. It is, in fact, our goal to make the entire MAGIC toolkits for all major chromosome arms. However, this task requires far longer than the 3-month turnaround time allowed for our revision. It requires molecular cloning, establishing, stabilizing, and verifying 24 different transgenic lines, and then functionally validating and comparing the transgenes in multiple tissues. The fly generation time alone means that simply generating and stabilizing the transgenes and fly-lines would take a minimum of 3 months, if all went well and optimally. The current pandemic makes the situation even worse. Both of our labs are small and resource-limited. The personnel on whom we rely to build these reagents currently have very limited accessibility to our partially-operating labs. Therefore, we expect that completing the 4 additional full kits requested will likely take much longer than we originally planned, well beyond 3 months.

We understand that it would have been ideal to have all MAGIC kits for all chromosome arms in this paper. Indeed, the original MARCM paper was an exemplar of that, providing complete reagents for all contingencies. But we are hoping that generating and validating one kit would be sufficient proof-of-principle for this paper, given the time and labor required to make all of the remaining kits. We plan to build the remaining kits in the near future, and will deposit them into stock centers as soon as they are ready and verified. Meanwhile, we have added in Table S2 the gRNAs we propose for the other chromosome arms, and will deposit the cloning vectors into Addgene. This will allow interested labs to start taking advantage of this new method by establishing transgenes for their own studies, while we build the kit for general use.

8. Line 169/170 - '…demonstrating the potential of MAGIC for clonal analysis of the function of natural alleles residing…'. The authors did not do any functional analysis of natural alleles. Please tone down the claims or at least discuss such issues in a more balanced manner.

Response: Thanks to the reviewer for this suggestion. We revised our text to tone down our claim.

9. The entire Discussion aims to sell the method, which in principle is fine. However, the entire Discussion should be written in a more balanced manner. Please do not discredit previous work based on FRT/Flp-mediated recombination. There are thousands of studies that exploit such technique and even once MAGIC is available for the community, many people will continue

using FRT/Flp systems for functional gene analysis. In fact, FRT sites have been inserted and reliably used on (almost) all chromosome arms. MAGIC currently is only enabling functional gene analysis on 2L.

Response: We apologize for giving the reviewer the impression of discrediting previous work based on FRT/Flp-mediated recombination – this was certainly not our intention. We revised the discussion in the hope that the reviewer and future readers will find our comparison of the two approaches to be objective and balanced.

Reviewer #2:
[identifies himself as Liqun Luo]

In this manuscript, the authors described a new method for mosaic analysis in Drosophila. Their method utilizes CRISPR/Cas9-induced double-strand breaks (DSBs) to induce mitotic recombination and thus can randomly produce homozygous clones in a heterozygous animal.

MAGIC offers two advantages. (1) The genetics is simpler compared to conventional approaches. (2) Because MAGIC does not require FRT sites, it will allow mosaic analysis for genes that cannot be analyzed by traditional approaches due to the position of existing FRTs. Thus, this new tool has potential to make mosaic analysis more doable and efficient, and even beyond the use in Drosophila.

I would be enthusiastic in supporting the publication of this manuscript if the authors can address the following issues in a revised manuscript:

1. The authors should compare the efficiency of gRNA-induced crossing-over with the efficiency of recombination mediated by FRT sites. This information will be critical for other researchers to determine if MAGIC is suitable for their study. We worry the efficiency could be markedly lower in MAGIC because DSB repair by non-homologous end joining (NHEJ) happens more frequently than homology-directed repair (HDR). In addition, when NHEJ occurs, it can mutate the gRNA target sites which prevents subsequent Cas9 cutting.

Response: We appreciate the reviewer's valid concern on the efficiency of MAGIC and thank him for suggesting comparisons between MAGIC and FRT/Flp. In the revised manuscript (Figure 3), we compared the efficiency of our 2L nMAGIC reagent in generating clones in the wing imaginal disc to that of FRT^{40A} using Cas9 and Flp lines, respectively, driven by the same enhancers. FRT^{40A} was chosen for the comparison because ubiquitously expressed fluorescent markers are available on FRT^{40A} chromosomes for easy identification and quantification of mosaic clones. Surprisingly, we found that MAGIC generated larger and more frequent clones than FRT/Flp. Although the low efficiency of the FRT/Flp method in these experiments may be due to the property of this particular FRT site, our results nevertheless demonstrate that MAGIC can be reliably used to generate mosaic clones in imaginal discs. Similarly, when we examined LOF phenotypes of *Sec5, Rab5, and Syx5* using SOP-Cas9 to induce mutant clones in da neurons, we were able to identify multiple clones in every larva. Although we did not compare the efficiencies of clone induction between MAGIC and FRT/Flp in da neurons, our experience suggests that MAGIC can be very usable in the nervous system as well, as long as appropriate gRNA target sites and Cas9 lines have been identified.

2. The author should demonstrate a temporally inducible way of making clones—such as using a heat shock promoter to drive Cas9. This is one of the most widely used ways to induce clones in the field. This is especially important in light of the caveat the authors raised in their

discussion: "For the cell type in question, an ideal Cas9 should be expressed in the precursor cells, as too early expression can mutate gRNA target sites prematurely and too late expression will lead to unproductive DSBs."

Response: We thank the reviewer for suggesting this experiment. We were fortunate to obtain from Dr. Tzumin Lee a HS-Cas9 line recently made by his lab. In our heat shock experiments using this line (Figure 3), we show that a single one-hour heat shock at 37°C at 72 hours after egg laying was sufficient to generate many clones in the wing imaginal disc, while larvae of the same genotype grown at 25°C showed almost no clones. Therefore, we conclude that MAGIC clones can be temporally induced.

3. Mosaic analysis technique papers in Drosophila have typically included resources that allow researchers to use the tools right away, rather than having to create the tools AND apply the tools. This will speed up the adoption of new techniques. The authors have produced tools for analysis of genes located on 2L, which covers only 20% of the genome. The paper will be greatly improved if the authors can also provide tools for other chromosomal arms. These will also serve to further validate the generality of the approach. I understand that this is a substantial amount of work (creating new transgenes) in particular during the pandemic, so I will leave it up to the journal editors to decide whether it is an option or a requirement.

Response: We appreciate the reviewer's comment and agree that it would be ideal to provide the full MAGIC kit to the fly community at the time of publication. It is, in fact, also our goal to make the entire MAGIC toolkits for all major chromosome arms. However, this task requires far longer than the 3-month turnaround time allowed for our revision. It requires molecular cloning, establishing, stabilizing, and verifying 24 different transgenic lines, and then functionally validating and comparing the transgenes in multiple tissues. Although the kits are intellectually straightforward to generate, and the fly manipulations are simple and standard too, the fly generation time alone means that simply generating and stabilizing the transgenes and fly-lines would take a minimum of 3 months, if all went well and optimally. The current pandemic makes the situation even worse. Both our labs are small and resource-limited. The personnel on whom we rely to build these reagents currently have very limited accessibility to our partially-operating labs. Therefore, we expect that completing the 4 additional full kits requested will likely take much longer than we originally planned, and well beyond 3 months.

We do understand that it would have been ideal to have all MAGIC kits for all chromosome arms in this paper. Indeed Dr. Luo's MARCM paper was an exemplar of that, providing complete reagents for all contingencies. But we are hoping that generating and validating one kit would be sufficient proof-of-principle for this paper, given the time and labor required to make all of the remaining kits. We plan to build the remaining kits in the near future, and will deposit them into stock centers as soon as they are ready and verified. Meanwhile, we have added in Table S2 the gRNAs we propose for the other chromosome arms and will deposit the cloning vectors into Addgene. This will allow interested labs to start taking advantage of this new method by establishing transgenes for their own studies, while we build the kit for general use.

Minor issues:

4. The statement on line 82 (a 50% chance…) is incorrect. G2-X (Fig. 1A top) and G2-Z (Fig. 1A, bottom) segregations are known to be unequal. There is a literature on this in Drosophila and in other organisms.

Response: Thanks for pointing out this error. We have included in the discussion that G2-X segregation is predominant in *Drosophila*, as shown in Beumer et al., Genetics 1998.

5. The illustration of MAGIC events should be kept consistent throughout the paper. Simplifying the sister chromatids to just one line can cause some confusions regarding when MAGIC event occurs (Figure 1B, 1F, 2A, 2B).

Response: We have changed these diagrams to keep the crossover schemes consistent.

6. In the legend for Figure 2C, what the grey boxes represent should be stated clearly.

Response: These boxes represent repeated sequences in the genome. We have added detailed annotations in the figure legend.

7. In figure 3, the total number of clones and the penetrance of the phenotype should be shown.

Response: Thanks for the suggestion. We added quantification and statistical data in the new Figure 4D to show the sample sizes and the penetrance of the phenotypes.

8. In line 181, "genetic modification" should be stated more explicitly as 'the requirement of FRT sites on the homologous chromosomes'.

Response: We thank the reviewer for the suggestion. We made the changes.

9. The authors stated that the ability to use MAGIC on DGRP wild-derived strains is one of its major advantages. However, we hoped that there can be more explanation for it. Specifically, the statement "it has been difficult to investigate the effect of homozygosity for alleles within these strains without being able to use available genetic tools" (line 212-213) is a bit confusing. Our understanding is that MAGIC cannot be directed crossed to those flies—either Cas9 or the MAGIC gRNA has to be combined with the allele first before the flies that produce mitotic recombination can be put together—to avoid keeping Cas9 and gRNA in the same fly across a generation. If this is the case, wouldn't MAGIC suffer the same constraint as other approaches?

Response: We realized that this important point was not explained adequately in our original text and we thank the reviewer for pointing it out. Theoretically, it is not desirable to keep Cas9 and gRNA in the same fly strain as target sites in Cas9-expressing tissues will be cut and subsequently mutated. This is especially important when Cas9 is expressed in the germline, as mutations will be passed to the progeny, rendering gRNAs ineffective. However, in practice, for Cas9 lines that do not have germline expression, including *hh-Cas9*, *zk-Cas9*, and *sop-Cas9* used in this study, we found that it is possible to keep MAGIC gRNAs and Cas9 transgenes in the same strain for multiple generations without affecting the efficiency of clone induction. Therefore, we can cross a strain that carries both a MAGIC gRNA and a Cas9 to DGRP files for mosaic analysis. We revised our discussion to clarify this point.

10. Drosophila has an unusual property that homologous chromosomes pair even in mitotic cell cycles, facilitating mitotic recombination. Most organisms do not have this property so it is unclear whether MAGIC would work. In their enthusiasm to state that MAGIC can be applied to other organisms, the authors should add this caveat.

Response: We thank the reviewer for pointing this out. We added discussions of this potential concern in our text.
