# Supplementary material for: Versatile CRISPR/Cas9-mediated mosaic analysis by gRNA-induced crossing-over for unmodified genomes
Source: PLoS Biol. 2021 Jan 14;19(1):e3001061. doi: 10.1371/journal.pbio.3001061 (PMC7837743; doi:10.1371/journal.pbio.3001061)
Supplement: Attachment — Submitted filename: MAGIC revis 2 R2R final.pdf [file pbio.3001061.s008.pdf]

Based on the reviews, we will probably accept this manuscript for publication, assuming that you will modify the manuscript to address the remaining points raised by the reviewers.

That is wonderful to hear; thank you!

IMPORTANT:

a) Please attend to the remaining requests from reviewer #2. Regarding this reviewer's point 1, the Academic Editor says that this can be addressed "by incorporating some additional possibilities in the discussion or by toning down the main statement."

Thanks for the advice on how to address point 1. We have followed it, as described below.

b) Many thanks for providing the data underlying the Figures in your supplementary S1_Data file. Please could you cite this in all relevant main and supplementary Figure legends? e.g. "The data underlying this Figure can be found in S1 Data."

Done.

c) We wonder if you could consider something more explicit for your title, to make your method's applications more evident? Perhaps "Using gRNA and CRISPR technology to facilitate mosaic analysis on a wide range of model organisms" or some such.

Since we only tested one species (though many wild genomes, as well as lab strains), we felt uncomfortable saying "wide range" of organisms, even though we expect MAGIC will work for a wide range, and non-models, as discussed in the text. We suggest the following title, which also implies the same kind of generality of system (as well as of genome-location). We happily welcome suggestions from you for alternative titles, if our suggestion is not of the generality needed or is otherwise not ideal.

Versatile CRISPR/Cas9-mediated mosaic analysis by gRNA-induced crossing-over for unmodified genomes

Reviewer #1:

The authors have addressed all concerns sufficiently. The newly added data and rewriting improved the manuscript.

Thank you!

Reviewer #2:
[identifies himself as Liqun Luo]

In the revised manuscript, the authors provided additional data to demonstrate the compatibility of MAGIC with heat shock and compared the efficiency of MAGIC with traditional FLP/FRT method. They have also incorporated textual changes that addressed most of our previous concerns.

Thank you!

However, we hoped that the authors can address following points regarding their new data:

(1) The results of comparing MAGIC with FLP/FRT is not completely satisfying. Firstly, the authors only compared one MAGIC construct (gRNA-40D2) with one FRT site (FRT40A). Without more thorough analysis using more examples, it is a bit of a stretch to generalize this observation to the conclusion that "Flp transgenes were much less efficient in generating clones than their Cas9 counterparts" (line 185-186). Secondly, no explanation is provided for why gRNA-40D2 is about 10-fold more efficient than FRT40A in the text. Thirdly, while zk-Cas9 generated fewer but larger clones and hh-Cas9 generated more but smaller clones, there is no significant differences between the clones generated by zk-FLP and hh-FLP. The authors should provide explanation for this.

We appreciate the reviewer's concern and apologize for giving the impression of generalizing the conclusion from this single set of comparisons. Our statement that "Flp transgenes were much less efficient in generating clones than their Cas9 counterparts" only described the results from the experiments in Figure 3 and was not intended to serve as a general conclusion. We have revised the description of the results to make it more specific and cautioned readers not to generalize the conclusion. Regarding the question about the difference between gRNA-40D2 and FRT40A in clone efficiency, we do not have an answer. Our speculation is that the FRT40A site is inefficient in this specific context. Based on our previous unpublished results, FRT40A seems to be less efficient than other FRT sites in imaginal discs and in larval peripheral neurons. We added this speculation to the text. Lastly, regarding the question about the potential difference between *zk-Flp* and *hh-Flp*, we did not observe statistical difference likely because the frequency of clones were too low in both cases. We added this reasoning to the text.

Minor issues:

(2) There is inconsistency between text and figure legend for which gRNA is used for MAGIC and FLP/FRT comparison experiment. In line 182, it says "gRNA-40A (nBFP)". But in legend line 643 it says "gRNA-40D2 (nBFP)".

We thank the reviewer for pointing out the typo in line 182. We have corrected it.

(3) In Figure 3D, shouldn't the heterozygous mother cell have blue and red mixture color instead of grey?

Thanks for the sensible suggestion. We have changed the color of the heterozygous mother cell to light purple.

(4) Based on the image examples, the branching number is also changed in Syx5 mutant comparing to wild type. Is this a common phenomenon for all Syx5 mutant?

Yes, the numbers of dendrite branches were greatly reduced in all *Syx5* mutant clones. We have added a graph to show the quantification of dendrite branch numbers in all genotypes (Figure 4E).
