# Supplementary material for: Versatile CRISPR/Cas9-mediated mosaic analysis by gRNA-induced crossing-over for unmodified genomes
Source: PLoS Biol. 2021 Jan 14;19(1):e3001061. doi: 10.1371/journal.pbio.3001061 (PMC7837743; doi:10.1371/journal.pbio.3001061)
Supplement: Attachment — Submitted filename: Dummy file.docx [file pbio.3001061.s006.docx]

Blank Page, please disregard
